# Genes Whose Gain or Loss of Function Changes Type 1, 2A, 2X, or 2B Muscle Fibre Proportions in Mice—A Systematic Review

**DOI:** 10.3390/ijms232112933

**Published:** 2022-10-26

**Authors:** Gabryela Kuhnen, Tiago Guedes Russomanno, Marta Murgia, Nicolas J. Pillon, Martin Schönfelder, Henning Wackerhage

**Affiliations:** 1 Department of Sports and Health Sciences, Technical University of Munich, 80809 Munich, Germany; 2 Max Planck Institute, Martinsried, 82152 Munich, Germany; 3 Department of Biomedical Sciences, University of Padova, Via Ugo Bassi, 58/B, 35131 Padua, Italy; 4 Department of Physiology and Pharmacology, Karolinska Institutet, 171 77 Stockholm, Sweden

**Keywords:** skeletal muscle fibre, muscle fibre proportions, myosin heavy chain

## Abstract

Adult skeletal muscle fibres are classified as type 1, 2A, 2X, and 2B. These classifications are based on the expression of the dominant myosin heavy chain isoform. Muscle fibre-specific gene expression and proportions of muscle fibre types change during development and in response to exercise, chronic electrical stimulation, or inactivity. To identify genes whose gain or loss-of-function alters type 1, 2A, 2X, or 2B muscle fibre proportions in mice, we conducted a systematic review of transgenic mouse studies. The systematic review was conducted in accordance with the 2009 PRISMA guidelines and the PICO framework. We identified 25 “muscle fibre genes” (*Akirin1*, *Bdkrb2*, *Bdnf*, *Camk4*, *Ccnd3*, *Cpt1a*, *Epas1*, *Esrrg*, *Foxj3*, *Foxo1, Il15*, *Mapk12*, *Mstn*, *Myod1*, *Ncor1*, *Nfatc1*, *Nol3*, *Ppargc1a*, *Ppargc1b*, *Sirt1*, *Sirt3*, *Thra*, *Thrb*, *Trib3*, and *Vgll2*) whose gain or loss-of-function significantly changes type 1, 2A, 2X or 2B muscle fibre proportions in mice. The fact that 15 of the 25 muscle fibre genes are transcriptional regulators suggests that muscle fibre-specific gene expression is primarily regulated transcriptionally. A reanalysis of existing datasets revealed that the expression of *Ppargc1a* and *Vgll2* increases and *Mstn* decreases after exercise, respectively. This suggests that these genes help to regulate the muscle fibre adaptation to exercise. Finally, there are many known DNA sequence variants of muscle fibre genes. It seems likely that such DNA sequence variants contribute to the large variation of muscle fibre type proportions in the human population.

## 1. Introduction

Human muscle fibres are up to 20 cm-long cells that produce force and heat [1]. Human muscle fibres are multinucleated cells, also termed syncytia, that develop because of myoblast fusion. Per millimetre of length, human muscle fibres have ≈50–250 myonuclei [2] and so we would expect 10,000–50,000 nuclei in a single, 20 cm-long human muscle fibre. Each skeletal muscle contains from hundreds up to many thousands of muscle fibres depending on its size. For example, the human vastus lateralis of ≈20-year-old males can contain between 393,000 and 903,000 muscle fibres [3].

The identification of different adult skeletal muscle fibres has evolved over time. From 1960–1967, researchers distinguished in-between fast white and slow red muscle fibres; from ≈1967–1975 type 1, 2A, and 2B fibres; and from ≈1986–1991 type 1, 2A, 2X, and 2B muscle fibres [4]. Different muscle fibres were first identified using enzyme assays [5,6] as well as histochemical and microscopic visualisation of ATPase activity after pre-incubation with an acidic or alkaline pH [7]. Myosin heavy chain isoforms (MHC), which determine the contraction velocity, were then visualised by immunocytochemistry utilising myosin heavy chain type 1, 2A, 2X, and 2B-specific antibodies [8], or, alternatively, with electrophoretic separation of myosin heavy chain isoforms [9]. Today, the concentrations of thousands of proteins in a single muscle fibre can be measured by first isolating single muscle fibres followed by an unbiased, proteomic analysis via mass spectrometry [10,11]. Currently, muscle fibres are primarily classified by the myosin heavy chain isoform(s) that they express. The major myosin heavy chain isoforms in humans are slow type 1 (gene *Myh7*), intermediate type 2A (gene *Myh*2), and fast 2X (gene *Myh1*) myosin [8]. Additionally, some rodent muscles—but not human muscles—contain the very fast type 2B myosin heavy chain protein (gene *Myh4*) [12]. In addition to fibres that exclusively (i.e., >90%) express one myosin heavy chain, there are so-called hybrid fibres that express two myosin heavy chain isoforms [13]. Moreover, embryonal (gene *Myh3*) and perinatal myosin (gene *Myh8*) heavy chains are expressed in embryonic, foetal, and regenerating muscle fibres [14].

In mammals, the distribution of muscle fibres varies greatly both intra- and inter-individually. For example, the red soleus is predominantly a type 1 slow twitch muscle, whereas the “whiter” rectus femoris contains faster type 2 muscle fibres [15]. In addition to this inter-muscle variability, muscle fibre percentages vary greatly between individuals. For example, an analysis of 418 human vastus lateralis biopsies reported 15–79% type 1 fibres, 13–77% type 2A fibres, and 0–44% type 2X fibres in the vastus lateralis and similarly large variations of metabolic enzyme activities [16]. Extreme muscle fibre compositions occur in athletes, with endurance athletes having typically a high percentage of type 1 and sprint and power athletes having a high percentage of type 2 fibres pin key locomotory muscles such as the vastus lateralis or gastrocnemius [17,18,19,20]. Extreme fibre type percentages are prerequisites for elite performance in both power/speed and endurance sports.

The distribution of skeletal muscle fibres depends both on an individual’s genetics (i.e., variations of the DNA sequence, or heritability) and environmental factors such as exercise training or diet as well as experimental variability, e.g., genetic manipulation on mice. Regarding humans, Bouchard and colleagues estimated that the variation in the proportion of type 1 fibres depends 45% on genetics, 40% on environmental factors, and 15% on experimental variability [21]. In addition to genetics, more innervation and contraction e.g., due to endurance training or fewer contractions e.g., due to denervation, reinnervation, or immobilisation alter fibre type-related gene expression and muscle fibre proportions. Specifically, exercise training promotes gene expression changes and minor fibre type shifts in a fast-to-slow direction. In their classic study, Gollnick, Saltin, and colleagues found that 5 months with four 1 h training sessions per week increased the percentage of slow twitch (i.e., type 1) fibres in the vastus lateralis non-significantly from 32% to 36% [22]. In the Heritage study, twenty weeks of endurance training increased the percentage of type 1 fibres by 3.5% and decreased the percentage of type 2X fibres by 5.4% [23]. Analysing the whole body of evidence suggests that exercise training over months can convert hybrid fibres (e.g., type 2X/2A) to pure (e.g., 2A) fibres and promote some (i.e., <10%) pure fibre conversions, e.g., from pure 2X to pure 2A fibres [24].

Whilst exercise interventions of less than a year result only in limited fibre transitions, it is unclear whether years of exercise training can cause major fibre type transitions where, e.g., >10% of fibres shift their type within a muscle. In contrast to exercise training, near complete fibre type transformations are caused by either denervation where fibres change in a slow-to-fast direction [25], innervation by a different motor neuron [26], or by chronic electrical low-frequency stimulation where many or almost all fibres change in a fast-to-slow direction [27,28].

Given that approximately half of the variation in human muscle fibre proportions is inherited, the question arises as to what genes and DNA sequence variants influence the proportions of muscle fibre types within a given muscle. Transgenic mouse models with germline mutations or injection of genetic constructs have helped to identify genes whose gain or loss of function significantly alters the proportions of type 1, 2A, 2X, or 2B muscle fibres. An important early study used gene expression, reporter assays, and pharmacological inhibition to identify the calcineurin-Nfat (nuclear factor of activated T-cells) pathway as a regulator of muscle fibre proportions [29,30]. Other transgenic mouse studies around the millennium showed that the overexpression of constitutively active *Ras* promotes a fast-to-slow fibre type change [31], and that the gain of function of the transcriptional co-activator Pgc-1α in muscle not only promotes mitochondrial biogenesis but also increases the number of type 1 fibres [32].

To date, the International Mouse Phenotyping Consortium (IMPC) has created and phenotyped over 5000 transgenic mouse strains [33]. However, the IMPC does not determine the muscle fibre composition of their mice, leaving a knowledge gap over what genes influence fibre type proportions in what muscles and by how much.

To address this question, we systematically searched the literature to identify genes whose gain or loss of function significantly alters the proportion of type 1, 2A, 2X, or 2B muscle fibres in mice. For simplicity, we refer to these genes, and to the proteins that they encode, from here onwards as “muscle fibre genes”. As the second step, we used online databases and re-analysed published datasets to answer questions such as: “In what tissues are muscle fibre genes expressed?” and “Does exercise alter the expression of muscle fibre genes or the phosphorylation of the proteins that they encode?”

## 2. Methods

### 2.1. Literature Search

We conducted a systematic review to identify genes whose gain or loss of function results in a statistically significant change in the percentage of at least one muscle fibre type or a significant change in myosin heavy chain isoform protein abundance in a mouse muscle. For the systematic literature review, we followed the 2009 preferred reporting items for systematic reviews and meta-analysis guideline (PRISMA [34]). The PICO framework [35] was also used to choose the key search word list, which was formulated as follows: population/problem; intervention; comparison; and outcome. Based on this, we used the following MeSH (medical subjects headings) listed in Table 1.

We used this search strategy to search the PubMed–Medline database for studies published up until November 2020. PubMed–Medline was used as the standard database due to its ability to match the full text of manuscripts with advance researching filtering and specific tools.

### 2.2. Inclusion and Exclusion Criteria

After the literature search, we screened all titles and abstracts to remove studies that did not report the effects of transgenesis on muscle fibre proportions in mice. After that, we selected studies from peer-reviewed journals, written in English, that reported muscle fibre type distribution and/or myosin heavy chain expression in gene-manipulated mouse models. We included studies that reported a measure of skeletal muscle fibre type numbers, percentages, or quantified myosin heavy chain isoform expression. We excluded studies as follows: (1) rat or in vitro study; (2) no transgenesis or double mutation; (3) miRNA manipulation; (4) mice with disease or pathological changes; (5) no statistically significant effect on muscle fibre distribution or myosin heavy chain isoform expression; (6) no comparison to wildtype or any other valid control; and (7) the gene manipulation resulted in a disease.

### 2.3. Data Collection, Extraction, and Analyses

From all included studies we extracted the following information: author; gene name; protein name; method of gene manipulation; animal acclimation; output measure; section of paper from where the data were extracted (figure or table); muscle(s) studied; number of fibre type or MHC expression in transgenic and control mice; difference between transgenic mice and control mice in percentage (this analysis was performed in Rstudio version 3.6.3, then exported to an excel sheet for further analysis); age of mice; mouse strain; additional measurements; and remarks. Additional columns for PubMed identificatory (PMID) paper, official gene name by the universal protein resource (UniProt, NCBI) and aliases regarding mice and humans. When the output measured appears only in bar graph or in stained muscle sections, we estimated the relative difference between transgenic and control group using ImageJ Software [36], and indicated this with an (*) on worksheet S2 in the supplementary Table S1.

### 2.4. Bioinformatic Analyses

After identifying muscle fibre genes, we conducted five bioinformatic analyses to add more information on the tissue of expression, common functions, and properties and their regulation by exercise, etc. More specifically the analyses investigated the following:

(1) To find out whether muscle fibre proteins are encoded we performed a String analysis (https://string-db.org/; RRID:SCR_005223 [37]).
(2) To identify common functions and properties of muscle fibre genes, we performed a ToppGene enrichment analysis (https://toppgene.cchmc.org/ [38]).
(3) To determine whether the skeletal muscle distribution and MHC expression genes are expressed specifically in skeletal muscle or elsewhere, we retrieved expression figures from the Genotype-Tissue Expression (GTEx; RRID:SCR_001618 [39]).
(4) To identify associations between muscle fibre genes and human phenotypes we searched the Genome-wide association studies (GWAS) catalogue (https://www.ebi.ac.uk/gwas/; RRID:SCR_012745).
(5) To find out whether muscle fibre genes change their expression after acute endurance exercise, resistance exercise, or in response to activity, we used the Meta-analysis of skeletal muscle response to exercise (MetaMEx) gene expression database to determine expression changes in muscle biopsies after acute endurance exercise, acute resistance exercise, and activity in health subjects (https://www.metamex.eu [40]). We also investigated whether muscle fibre proteins become phosphorylated or dephosphorylated after exercise. For this, we retrieved supplementary data from two phospho-proteome studies. Study 1 investigated protein phosphorylation changes after a single bout of high intensity training in human muscles [41]. Study 2 investigated protein phosphorylation in mouse skeletal muscle after electrically evoked maximal-intensity contractions [42].

More complete data of the above analyses are in the supplementary data.

## 3. Results and Discussion

### 3.1. Results

**Figure 1:**
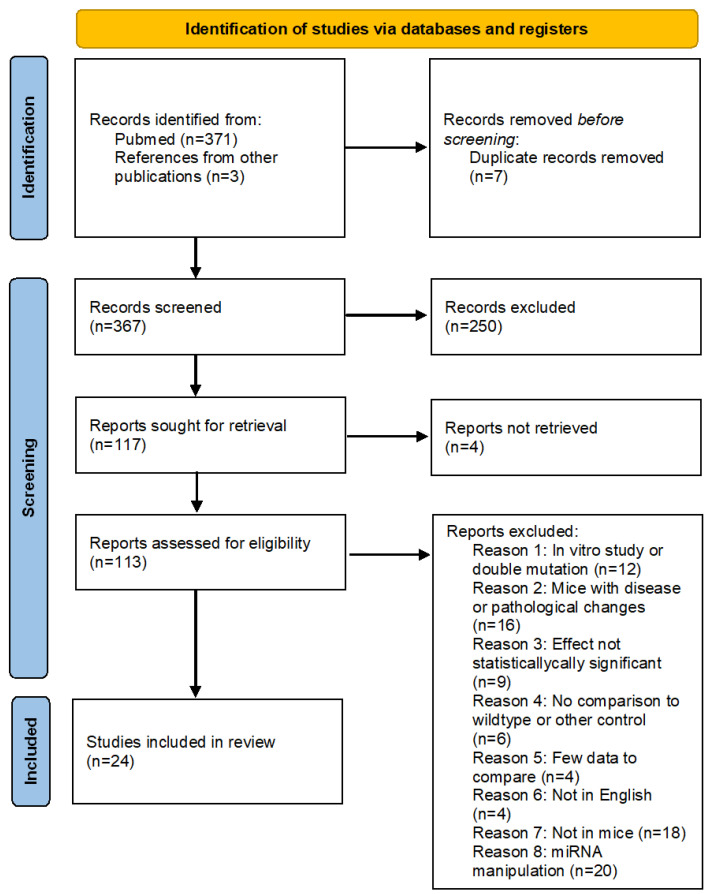
PRISMA flow chart of the study research methodology used on the databases. See also supplemental data worksheet S1.

The initial PubMed–Medline search yielded 371 studies published before November 2020. We identified another three studies through other sources. After reading titles and abstracts, we excluded 261 manuscripts and 113 articles remained. We then read the full text of these articles and excluded more articles based on our inclusion and exclusion criteria. In the end, 24 publications were analysed quantitatively. The PRISMA flowchart in Figure 1 summarises the search and subsequent selection of the publications analysed in this study.

#### Genes Whose Gain or Loss of Function Significantly Changes Muscle Fibre Distribution in Mice

Overall, we identified 25 genes whose gain or loss of function significantly changed the percentages of type 1, 2A, 2X, or 2B muscle fibres or myosin heavy chain abundance in at least one muscle in mice. A summary of the studies included, and some of their main findings, is given in Table 2.

**Figure 2:**
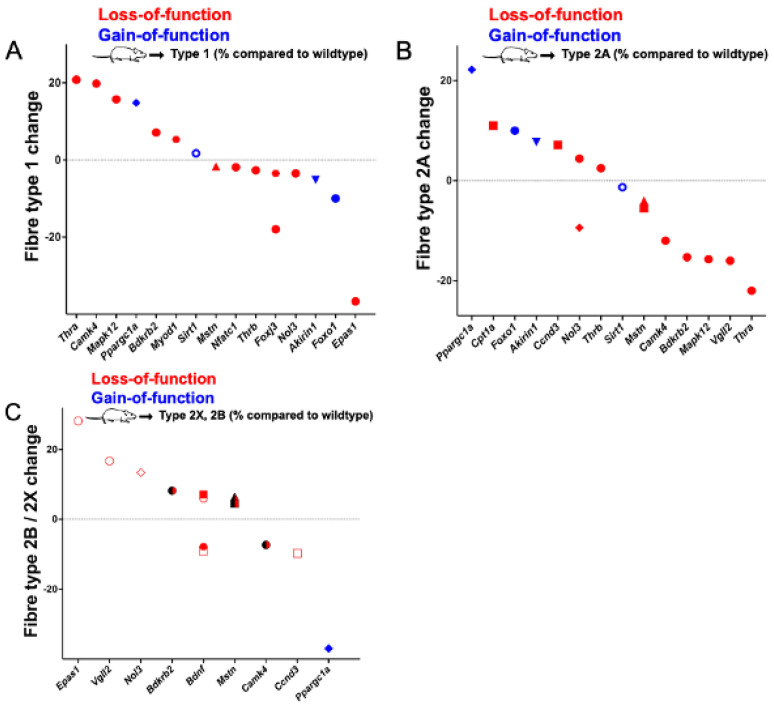
Genes whose gain or loss of function significantly changes the percentages of type 1 (**A**), type 2A (**B**), or type 2X/B (**C**) fibres when compared to wildtype or control mice. Red symbols: loss-of-function; blue symbols: gain-of-function. ● Soleus; ⬣ extensor digitorum longus (EDL); ◆ plantaris; ◼ tibialis anterior; ▲ biceps femoris; ▼ quadriceps; and **◯** gastrocnemius. In (**C**), filled symbols refer to 2X muscle fibres and symbols without filling indicated 2B fibres. Note that the “**◯**”-marked data in (**A**,**B**) were reported as statistically significant even though they are close to “0” (i.e., no change).

**Figure 3:**
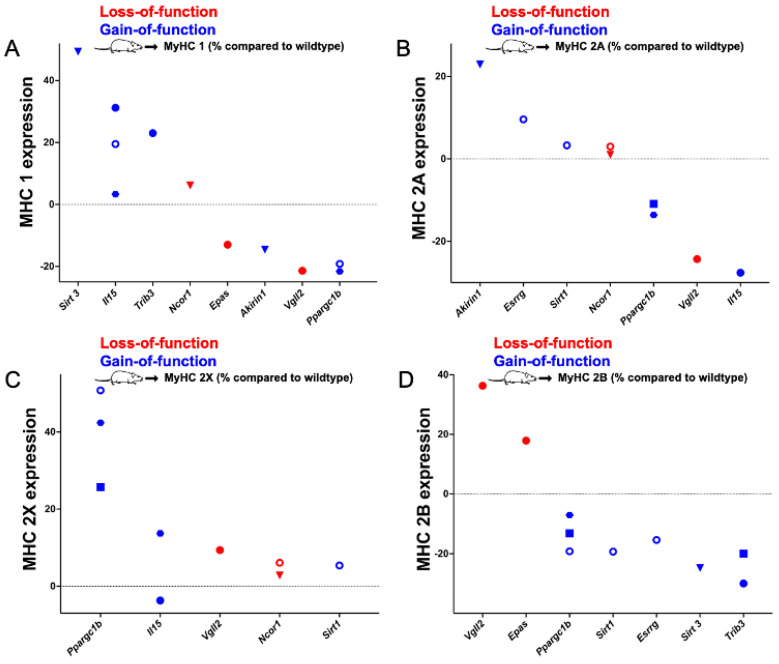
Genes whose gain or loss of function significantly changes the relative expression of type 1 (**A**), type 2A (**B**), type 2X (**C**), or type 2B (**D**) myosin heavy chain (MHC) isoforms when compared to wildtype or control mice. Red symbols: loss-of-function; blue symbols: gain-of-function. ● Soleus; ⬣ extensor digitorum longus (EDL); ◼ tibial anterior; ▼ quadriceps; and **◯** gastrocnemius.

Genes whose gain or loss of function significantly changed the percentage of type 1, 2A, or 2X fibres are presented in Figure 2 and the genes that affect myosin heavy chain isoform expression are shown in Figure 3.

Specifically, we identified 13 genes (*Bdkrb2*, *Bdnf*, *Camk4*, *Ccnd3*, *Cpt1a*, *Foxj3*, *Mapk12*, *Mstn*, *Myod1*, *Nfatc1*, *Nol3*, *Thra*, and *Thrb*) whose loss of and two genes whose gain of function (*Foxo1, Ppargc1a*) significantly changed the proportions of at least one muscle fibre type. Additionally, we identified one gene whose knockout (*Ncor1*) and five genes whose gain of function (*Esrrg*, *Il15*, *Ppargc1b*, *Sirt3*, and *Trib3*) significantly altered the expression of at least one myosin heavy chain isoform in mice. Moreover, loss of function of two genes (*Epas1* and *Vgll2*) and the gain of function of two other genes (*Akirin1* and *Sirt1*) significantly altered both the proportions of at least one muscle fibre type and significantly changed the expression of at least one myosin heavy chain isoform. The effect sizes of the gene manipulations range from a reduction in a fibre type by 37% (*Ppargc1a*, Plantaris type 2B fibres) to a gain of 28% of a single fibre type (*Epas1*, soleus type 2B fibres). Genes whose knockout affected more than one fibre type in the soleus are *Bdkrb2*, *Camk4*, *Mpak12*, *Nol3*, *Thra*, and *Thrb*. The muscle fibre genes *Bdnf*, *Ccnd3*, and *Mstn* affected muscle fibre distribution in the tibialis anterior, *Bdnf* in the extensor digitorum longus, and *Nol3* in the plantaris. In addition, *Foxo1* affected more than one fibre type in the soleus, and, similarly, *Ppargc1a* in the plantaris. *Ncor1* knockout affected the myosin heavy chain expression in the gastrocnemius and quadriceps. On the other hand, overexpression of *Esrrg*, *Il15*, *Ppargc1b*, and *Sirt3* changed muscle fibre proportions in the gastrocnemius; *Il15* and *Trib3* in the soleus; *Il15* and *Ppargc1b* in the extensor digitorum longus; and *Ppargc1b* and *Trib3* in the tibialis anterior.

Next, we used the list of 25 muscle fibre genes to answer direct research questions through further bioinformatic analyses. Information on the searches and detailed results can be found in the supplemental data.

(1) Do muscle fibre proteins interact, and do muscle fibre genes share common functional features?

**Figure 4:**
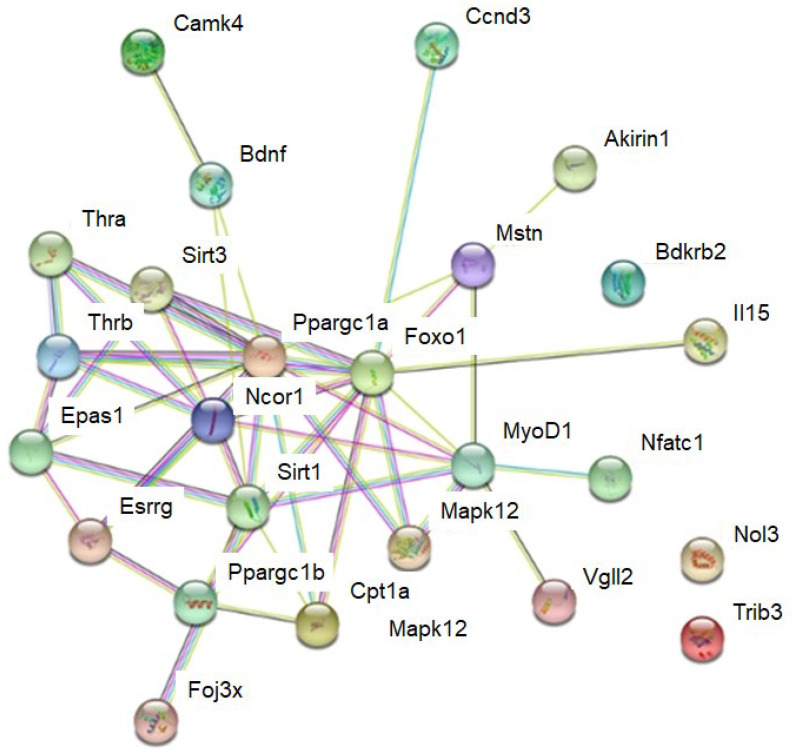
String analysis for interactions between muscle fibre proteins. Lines between proteins indicate evidence for interaction. See supplemental data S5 for more detail.

To answer this question, we completed a String protein interaction analysis [37] and a ToppGene enrichment analysis [38]. The String analysis suggests interactions in and between muscle fibre genes. Clusters of muscle fibre genes included a cluster of genes that encoded thyroid (i.e., the expression of all MYH genes that respond to the thyroid hormone [66]) and oestrogen hormone receptors (*Thra*, *Thrb*, and *Essrg*). Further, there were also a cluster with the transcriptional co-factors and transcription factors *Ppargc1a*, *Ppargc1b*, *Vgll2*, *Foxo1*, *Myod1*, and *Nfatc1*; the sirtuins *Sirt1* and *Sirt3*; and a cluster of the circulating factors *Mstn* and *Bdnf*, as well as the kinases *Mapk12* and *Camk4* (Figure 4).

We also used a ToppGene functional enrichment analysis to identify common features and functions among the muscle fibre genes identified. Specifically, we found 15 muscle fibre genes that regulate gene transcription (*Foxo1*, *Ppargc1a*, *Ncor1*, *Ppargc1b*, *Thra*, *Thrb*, *Akirin1*, *Trib3*, *Nfatc1*, *Foxj3*, *Vgll2*, *Myod1*, *Epas1*, *Sirt1*, and *Esrrg*), 5 genes that regulate muscle adaptation to contractile activity (see below), loading conditions, substrate supply, and environmental factors. Of the 25 muscle fibre genes, 13 genes regulate cellular responses to hormones (*Ccnd3*, *Foxo1*, *Ppargc1a*, *Ncor1*, *Ppargc1b*, *Thra*, *Thrb*, *Mstn*, *Myod1*, *Bdnf*, *Sirt1*, and *Esrrg*) as well as 6 genes that are linked to energy metabolism, i.e., in the form of mitochondrial biogenesis (*Foxo1*, *Ppargc1a*, *Ppargc1b*, *Camk4*, *Sirt3*, and *Sirt1*) (see supplemental data worksheet S7 for more detail).

(2) In what human tissues are muscle fibre genes expressed?

**Figure 5:**
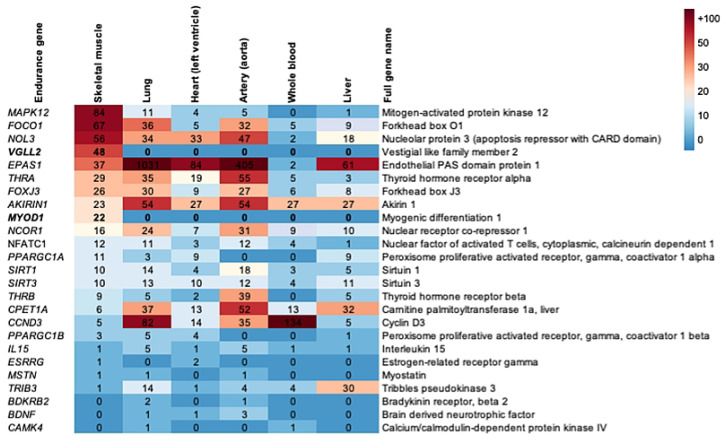
Heatmap illustrating the expression of muscle fibre genes in different human tissues in transcript per million (TPM) and in the order of highest expression in skeletal muscle. See also supplemental data worksheet S6.

To find out whether muscle fibre genes are primarily expressed in skeletal muscle or elsewhere, we retrieved human gene expression data from the Genotype-Tissue Expression (GTEx) Project website (https://gtexportal.org/home [39]). This analysis revealed that two of the muscle fibre genes, *Vgll2* and *MyoD1*, are exclusively expressed in skeletal muscle. Moreover, *Mapk12*, *Foxo1*, and *Nol3* are most expressed in the human skeletal muscle, but they are also expressed elsewhere in the body (Figure 5).

(3) Are muscle fibre genes regulated in response to exercise or inactivity?

**Figure 6:**
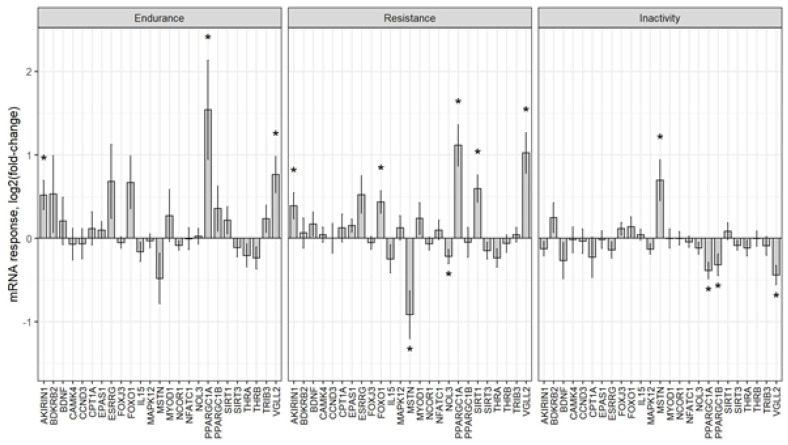
Meta−analysed expression change data of muscle fibre genes after acute bouts of endurance exercise, resistance exercise, and inactivity (“*” indicates FDR < 0.05). See supplemental data worksheet S4 for more detail.

To systematically investigate whether muscle fibre genes are regulated by exercise, we retrieved from the MetaMEx website (https://www.metamex.eu/ [40]) meta-analysed human muscle fibre gene expression data, which compared pre and post exercise as well as inactivity data (Figure 5. Worksheet S4 in Supplementary Table S1 [40]). To find out whether muscle fibre proteins are phosphorylated, and whether they become phosphorylated or dephosphorylated after a bout of human exercise, we also retrieved published phosphoproteomics data [41] to analyse (see supplemental data worksheet S8 for more detail). The gene expression analysis identified *PPARGC1A*, which encodes the mitochondrial biogenesis regulator Pgc-1α; further, it also identified *VGLL2* as the gene that roughly doubles its expression after acute bouts of endurance or resistance exercise in the human vastus lateralis muscle, and that which decreases its expression in inactive muscles. *EPAS1*, which encodes a hypoxia-induced transcription factor, also increases its expression after a bout of endurance and resistance exercise but decreases in response to inactivity. Conversely, *MSTN* expression decreases after a bout of endurance and resistance exercise but increases in response to inactivity. The expression changes in all muscle fibre genes in response to acute endurance exercise, acute resistance exercise, and inactivity are shown in Figure 6.

This reveals that muscle fibre genes such as *PPARGC1A* and *VGLL2* increase their expression, whereas *MSTN* decreases its expression especially after a bout of endurance exercise. However, the opposite is true for inactivity.

When analysing muscle protein phosphorylation, we found that *Vgll2* Ser261 phosphorylation increased by 30% after maximal muscle contractions in mice (*p* = 0.07) [42]. In addition, *FOXO1*, *MAPK12*, *NOL3*, *NCOR1*, and *SIRT1* were detected as phosphorylated proteins in human muscle after a single high-intensity exercise bout. However, of these muscle fibre proteins, only *MAPK12* Ser362 phosphorylation increased by more than 1.5-fold (*p* < 0.05) [41]. Collectively, this suggests that several muscle fibre genes are regulated in response to acute endurance or resistance exercise or inactivity.

(4) What is known about sequence variability of the muscle fibre genes in human exome?

Human fibre type distribution in muscles vary in the human population and this is partially, ≈45%, explained by genetics [21]. To determine the frequency of human DNA sequence variants of muscle fibre genes, we retrieved exome sequence data for 60,706 humans [67]. The analysis of this data revealed that each muscle fibre gene had on average 160 missense, 3 loss-of-function, and 87 synonymous DNA variants (see supplemental data worksheet S9 for more detail). For *BDKRB2*, *CCND3*, *NOL3*, *THRA*, *NCOR1*, and *EPAS2* homozygous loss-of-function DNA variants are reported [67]. Together, this suggests that exome sequence variability of human muscle fibre genes could at least partially explain the currently poorly explained variability of muscle fibre genes. Moreover, we also searched the term “muscle fibre genes” in the GWAS catalogue (https://www.ebi.ac.uk/gwas/) to find out whether these genes are associated with similar physiological or pathological phenotypes that could be linked to muscle fibre alterations. However, we did not find any systematic pattern (see supplemental data worksheet S3 for more detail).

### 3.2. Discussion

By conducting a systematic review, we have identified 25 genes whose gain or loss of function significantly changes muscle fibre percentages, or the abundance of myosin heavy chain isoforms in mouse muscles. This confirms that muscle fibre type proportions are a polygenic trait. The effect sizes of the mutated, individual genes range from a 37% reduction in plantaris type 2B fibres (*Ppargc1a*) to a 28% increase in type 2B fibres in the soleus muscle (*Epas1*). There is no muscle fibre gene whose manipulation results in a transformation of all muscle fibres to another muscle fibre type. There are known human DNA sequence variants for all muscle fibre genes, and this could be useful to identify in future DNA sequence variants that have a measurable effect size on human muscle fibre distribution.

The fact that 15 of the 25 muscle fibre genes (*Foxo1*, *Ppargc1a*, *Ncor1*, *Ppargc1b*, *Thra*, *Thrb*, *Akirin1*, *Trib3*, *Nfatc1*, *Foxj3*, *Vgll2*, *Myod1*, *Epas1*, *Sirt1*, and *Esrrg*), are transcriptional regulators suggests that muscle fibre-specific gene expression is primarily regulated transcriptionally. How these transcription factors and co-factors combine to regulate the coordinated expression of hundreds of muscle fibre-specific genes—in either a binary (i.e., an on/off regulation such as the case for *Myh* genes in pure fibres) or graded fashion (i.e., a higher/lower expression, e.g., mitochondrial genes)—is incompletely understood. Generally, the identity of cells, such as in muscle fibre identity, is often regulated by super-enhancers. Such super-enhancers are groups of enhancers bound by master transcription factors such as *Brd4* and *Med1* that compartmentalise DNA and its genes, allowing a coordinated regulation of the expression of target genes [68,69]. Consistent with this, the muscle-specific knockout of the super-enhancer-associated factor *Med1* resulted in increased expression of *Myh7* (type 1 myosin heavy chain), *Myh2* (type 2A), as well as myoglobin and metabolic gene mRNAs. This suggests a coordinated regulation of muscle fibre identity-related genes [70]. This is consistent but does not proof that *Med1* targets a fibre identity-regulating super enhancer. 

Super enhancers are often associated with long intergenic non-coding RNA (lincRNAs) [71]. In relation to this, linc-MYH was identified as a lincRNA in “fast fibre” nuclei that inhibits slow-type enhancers and promotes fast/glycolytic muscle fibre gene expression [72]. The regulation of multiple fibre identify associated genes is consistent with a fast/glycolytic fibre super enhancer. Finally, Gunderson and colleagues have investigated the epigenetics of purified myonuclei from the predominantly fast twitch extensor digitorum longus and the slow twitch soleus mouse muscles [73]. They found an overrepresentation of binding sites for *Mef2C*, *Nfatc2*, and *Ppara* in the soleus and of *MyoD1* and *Sox1* in the EDL. There is knowledge of multiple, muscle fibre type identify regulating transcription factors, the likely existence of a fibre type-regulating super enhancer [72], and methods allowing to analyse transcriptional regulation of nuclei with a fast/glycolytic or slow/oxidative identity [73]. These should enable future researchers to find out why more than ten transcription factors can regulate sets of genes associated with a fast/glycolytic or slow/oxidative muscle fibre identity.

Motor neuron activity and contractions are the main stimuli for muscle fibre-specific gene expression and for muscle fibre transitions. Consistent with this, our analysis reveals several muscle fibre genes that are regulated by exercise or immobilisation. A key mode of regulation for adaptation to muscle contractions and exercise is transcriptional regulation. For example, acute human endurance or resistance exercise increases the expression of muscle fibre genes such as *PPARGC1A* and *VGLL2* but decreases the expression of *MSTN* [40]. In contrast, inactivity decreases the expression of muscle fibre genes such as *VGLL2* but increases the expression of *MSTN* [40]. A second mode of regulation is post-translational modification of muscle fibre proteins especially by phosphorylation. One example for this is the 1.5-fold increased phosphorylation of *MAPK12* at Ser362 [41]. Finally, muscle fibre proteins can also be regulated by translocation. Prime examples of this are *Nfat* transcription factors. For example, *Nfatc1* is more nuclear in slower type 1 than faster type 2 fibres and, therefore, translocates to the nucleus in response electrical stimulation [74]. Whether and how this drives gene expression that is related to muscle fibre identity, however, is poorly understood.

Finally, all muscle fibre genes are affected by human DNA sequence variation. This ranges from rare, homozygous knockouts of the whole gene (such as in the case of *MSTN* [75]) to single nucleotide polymorphisms that occur in all muscle fibre genes [67], such as *Bdnf*, *Camk4*, and *Ccnd3*. Given that the distribution of muscle fibres is ≈50% inherited [21], and given that we know few muscle fibre-proportion varying DNA sequence variants, the list of muscle fibre genes could be used to find out whether DNA sequence variants of muscle fibre genes occur at a higher or lower frequency in, e.g., elite sprinters or endurance athletes with often extreme fibre type proportions when compared to the sedentary population. Such a targeted, genetic analysis might help to uncover the genetic causes of the variability of muscle fibre proportions in the human population.

The first limitation of this study is that the choice of the targeted genes is subjective, that not all genes in the genome have been manipulated and that, e.g., the IMPC phenotyping pipeline does not include muscle fibre typing. A second limitation is that it is impossible to determine whether gene manipulation affects development, e.g., by blocking the slow-to-fast switch that occurs after birth in the fast muscles or is instead related to adult muscle fibre plasticity by, e.g., promoting a fast-to-slow twitch [4]. A third limitation is the strict inclusion and exclusion criteria used that included the use of “statistical significance” as an inclusion criterion. For example, the manipulation of genes such as *Actn3* results in nearly significant fibre type changes (e.g., *p* = 0.051 in the spinalis) [76] and this is an issue because of the limited *p*-values [77] used. Further, as a systematic review requires clearcut inclusion and exclusion criteria, and due to the fact that we have chosen statistical significance as an inclusion criterion, this study cannot, therefore, be considered to be a systematic review. Fourth and finally, some publications suggest fibre type shifts based on mRNA expression alone and these were not included. Examples are transgenic mice with transgenes of *p43* [78] and *Med1* [70].

## 4. Conclusions

In summary, we detected 25 genes whose gain or loss of function alters muscle fibre proportions in mice. Given that the 25 genes causally affect fibre proportions, functionally relevant DNA sequence variants that are related to these genes should affect human muscle fibre type proportions, provided that the function of these genes is conserved between mice and men. Together with a list of muscle hypertrophy genes [79], endurance genes [80], and glucose uptake genes [81] this additional list of causative genes should help to identify causative DNA variants that influence human sport and exercise-related traits [82] as well as human health—as many exercise-related traits are predictors of health.

## Figures and Tables

**Table 1:** Search Strategy used.

Database	Search Formula
Medline (via PubMed)	(mice OR “mouse” OR “mouse transgenic” OR “mice transgenic” OR “mouse knockout” OR “mice knockout” OR “mouse model” OR “mice model” OR “mice overexpressed” OR “mouse overexpressed”) AND (“gene expression” OR “gene knockout” OR “gene overexpression” OR “gene knock in” OR “gene transfer techniques” OR “gene deletion”) AND (“muscle fiber distribution” OR “muscle fiber fast twitch” OR “muscle fiber slow twitch” OR “muscle fiber type I” OR “muscle fiber type II” OR “oxidative muscle” OR “oxidative fiber” OR “glycolytic muscle” OR “glycolytic fiber”)

**Table 2:** Summary of included studies. Main characteristics and outcome.

Study	Gene	Knockout or Overexpression	Fibre Type Analysis Procedure	Output Measure	Outcome (Relative to Wildtype)
[43]	*Akirin1*	Knockout	MHC immunofluorescence MHC analysis	Quadriceps Type 1	↓
				Quadriceps Type 2A	↑
				Quadriceps *Myh7* 1	↓
				Quadriceps *Myh2* 2A	↑
[44]	*Bdkrb2*	Knockout	ATPase staining	Soleus % type 1	↑
				Soleus % type 2A	↓
				Soleus % type Intermediary	↑
[45]	*Bdnf*	Knockout	MHC immunohistochemistry	TA % type 2X	↑
				TA % type 2B	↓
				EDL % type 2X	↑
				EDL % type 2B	↓
[46]	*CamK4*	Knockout	MHC immunofluorescence	Soleus % type 1	↑
				Soleus % type 2A	↓
				Soleus % type other	↓
[47]	*Ccnd3*	Knockout	MHC immunostaining	TA % myofiber type 2A	↑
				TA % myofiber type 2B	↓
[48]	*Cpt1a*	Conditional knockout	MHC immunostaining	TA % type 2A	↑
[49]	*FoxJ3*	Knockout	ATPase staining	EDL % type 1	↓
				Soleus % type 1	↓
[50]	*Foxo1*	Overexpression	ATPase staining	Soleus number type 1	↓
				Soleus number type 2	↑
[51]	*Mapk12*	Knockout	MHC immunostaining	Soleus % type 1	↑
				Soleus % type 2A	↓
[52]	*Mstn*	Knockout	MHC immunostaining	Biceps femoris % type 1	↓
				Biceps femoris % type 2A	↓
				Biceps femoris % type 2B/X	↑
				TA % type 2A	↓
				TA % type 2B/X	↑
[53]	*MyoD1*	Knockout	MHC immunohistochemistry	EDL % Type 1	↑
[54]	*Nfatc1*	Conditional knockout	ATPase staining	Soleus number Type 1	↓
[55]	*Nol3*	Knockout	Immunofluorescence MHC	Soleus % type 1	↓
				Soleus % Type 2A	↑
				Plantaris % Type 2A	↓
				Plantaris % Type 2B	↑
[32]	*Ppargc1a*	Overexpression	ATPase staining	Plantaris number Type 1	↑
				Plantaris number Type 2A	↑
				Plantaris number Type 2B	↓
[56]	*Thra*	Knockout	ATPase staining	Soleus % Type 1	↑
				Soleus % Type 2A	↓
	*Thrb*			Soleus % Type 1	↓
				Soleus % Type 2A	↑
[57]	*Epas1*	Conditional Knockout	MHC analysis	Soleus number type 1	↓
				Soleus number type 2B	↑
				Soleus MHC 1	↓
				Soleus MHC 2B	↑
[58]	*Esrrg*	Conditional overexpression	MHC gene expression analysis	Gastrocnemius MHC 2A	↑
				Gastrocnemius MHC 2B	↓
[59]	*Il15*	Conditional overexpression	MHC gene expression analysis	Soleus MHC 1	↑
				Soleus MHC 2A	↓
				Soleus a MHC 2X	↓
				EDL MHC 1	↑
				EDL MHC 2X	↑
				Gastrocnemius MHC 1	↑
[60]	*Ncor1*	Conditional knockout	MHC gene expression analysis	Gastrocnemius MHC 2A	↑
				Gastrocnemius MHC 2X	↑
				Quadriceps MHC 1	↑
				Quadriceps MHC 2A	↑
				Quadriceps MHC 2X	↑
[61]	*Ppargc1b*	Conditional overexpression	MHC gene expression analysis	Gastrocnemius MHC 1	↓
				Gastrocnemius MHC 2X	↑
				Gastrocnemius MHC 2B	↓
				EDL MHC 1	↓
				EDL MHC 2A	↓
				EDL MHC 2X	↑
				EDL MHC 2B	↓
				TA MHC 2A	↓
				TA MHC 2X	↑
				TA MHC 2B	↓
[62]	*Sirt3*	Conditional overexpression	Western blot	Quadriceps MHC 1	↑
				Quadriceps MHC 2B	↓
[63]	*Trib3*	Conditional overexpression	Electrophorese of MHC	Soleus MHC 1	↑
				Soleus MHC 2B	↓
				TA MHC 2B	↓
[64]	*Vgll2*	Knockout	qPCR MHC analysis	Soleus number type 2A	↓
				Soleus number type 2B	↑
				Soleus Myh7 1	↓
				Soleus Myh2 2A	↓
				Soleus Myh1 2X	↑
				Soleus Myh4 2B	↑
[65]	*Sirt1*	Conditional overexpression	ATPase staining and PCR MHC analysis	Gastrocnemius % Type 1	↑
				Gastrocnemius % Type 2	↓
				Gastrocnemius MHC 2A	↑
				Gastrocnemius MHC 2X	↑
				Gastrocnemius MHC 2B	↓

Legend ↑ Significantly increased proportion, concentration, or expression when compared to control. ↓ Significantly decreased proportion, concentration, or expression when compared to control. For more detail, see also Table S2 in the supplemental data.

## Data Availability

Data are available in the supplementary data file.

## Supplementary Materials

The following supporting information can be downloaded at: https://www.mdpi.com/article/10.3390/ijms232112933/s1.

## Abbreviations

*Actn3*	Actinin alpha 3
*Akirin1*	Akirin 1
*Bdkrb2*	Bradykinin receptor 2
*Bdnf*	Brain derived neurotrophic factor
*Brd4*	Bromodomain containing 4
*Camk4*	Calcium-/calmodulin-dependent protein kinase IV
*Ccnd3*	Cyclin D3
*Cpt1a*	Carnitine palmitoyltransferase 1A
EDL	Extensor digitorum longus
*Epas1*	Endothelial PAS domain protein 1
*Esrrg*	Oestrogen related receptor gamma
*Foxj3*	Forkhead box J3
*Foxo1*	Forkhead box O1
GTEx	Genotype-Tissue Expression
GWAS	Genome Wide Association Studies
*Il15*	Interleukin 15
IMPC	International Mouse Phenotyping Consortium
lincRNAs	long intergenic non-coding
*Mapk12*	Mitogen-activated protein kinase 12
*Med1*	Mediator complex subunit 1
*Mef2C*	Myocyte enhancer factor 2C
MeSH	Medical Subjects Heading
MetaMEx	Meta-analysis of Skeletal Muscle Response to Exercise
MHC	Myosin Heavy Chain
*Mstn*	Myostatin
*Myh1*	Myosin heavy chain 1; encodes myosin 2X protein
*Myh2*	Myosin heavy chain 2; encodes myosin 2A protein
*Myh4*	Myosin heavy chain 4; encodes myosin 2AB protein
*Myh7*	Myosin heavy chain 7; encodes myosin type 1 protein
*Myod1*	Myogenic differentiation 1
NCBI	National Center for Biotechnology Information
*Ncor1*	Nuclear receptor corepressor 1
*Nfatc1*	Nuclear factor of activated T cells 1
*Nfatc2*	Nuclear factor of activated T cells 2
*Nol3*	Nuclear protein 3
PICO	Population/Problem, Intervention, Comparison, Outcome
PMID	PubMed identificatory
*Ppargc1a*	Peroxisome proliferative activated receptor, gamma, coactivator 1 alpha
*Ppargc1b*	Peroxisome proliferative activated receptor, gamma, coactivator 1beta
*Ppara*	Peroxisome proliferator activated receptor alpha
PRISMA	Preferred Reporting Items for Systematic Review and Meta-Analysis Guideline
*Sirt1*	Sirtuin 1
*Sirt3*	Sirtuin 3
*Sox1*	SRY (sex determining region Y)-box 1
*Thra*	Thyroid hormone receptor alpha
*Thrb*	Thyroid hormone receptor beta
TPM	Transcript per million
*Trib3*	Tribbles pseudokinase 3
Uniprot	Universal Protein Resources
*Vgll2*	Vestigial like family member 2
